# Repeated Intravaginal Inoculation of Zika Virus Protects Cynomolgus Monkeys from Subcutaneous Superchallenge

**DOI:** 10.3390/ijms232214002

**Published:** 2022-11-13

**Authors:** Maya Shofa, Tomotaka Okamura, Emiko Urano, Yoshiharu Matsuura, Yasuhiro Yasutomi, Akatsuki Saito

**Affiliations:** 1 Department of Veterinary Science, Faculty of Agriculture, University of Miyazaki, Miyazaki 889-2192, Japan; 2 Graduate School of Medicine and Veterinary Medicine, University of Miyazaki, Miyazaki 889-1692, Japan; 3 Tsukuba Primate Research Center, National Institutes of Biomedical Innovation, Health and Nutrition, Ibaraki 305-0843, Japan; 4 Center for Infectious Disease Education and Research, Osaka University, Osaka 565-0871, Japan; 5 Research Institute for Microbial Diseases, Osaka University, Osaka 565-0871, Japan; 6 Department of Molecular and Experimental Medicine, Mie University Graduate School of Medicine, Mie 514-8507, Japan; 7 Center for Animal Disease Control, University of Miyazaki, Miyazaki 889-2192, Japan

**Keywords:** Zika virus, intravaginal infection, superchallenge, neutralizing antibody

## Abstract

Zika virus (ZIKV) outbreaks in Central and South America caused severe public health problems in 2015 and 2016. These outbreaks were finally contained through several methods, including mosquito control using insecticides and repellents. Additionally, the development of herd immunity in these countries might have contributed to containing the epidemic. While ZIKV is mainly transmitted by mosquito bites and mucosal transmission via bodily fluids, including the semen of infected individuals, has also been reported. We evaluated the effect of mucosal ZIKV infection on continuous subcutaneous challenges in a cynomolgus monkey model. Repeated intravaginal inoculations of ZIKV did not induce detectable viremia or clinical symptoms, and all animals developed a potent neutralizing antibody, protecting animals from the subsequent subcutaneous superchallenge. These results suggest that viral replication at mucosal sites can induce protective immunity without causing systemic viremia or symptoms.

## 1. Introduction

Zika virus disease is caused by Zika virus (ZIKV), which is a single-stranded RNA virus of the *Flaviviridae* family, genus *Flavivirus*. ZIKV was first identified in a rhesus macaque from Uganda in 1947 [1]. ZIKV is mainly transmitted by *Aedes* mosquitoes, including *A. aegypti* and *A. albopicus*. While most ZIKV infections are asymptomatic, the observed symptoms include mild fever, headache, red eyes, arthralgia, conjunctivitis, and rashes. Moreover, a link between ZIKV infection and Guillain-Barré syndrome (GBS) has been documented [2,3]. Currently, no vaccine or specific antiviral drug is available to prevent or treat ZIKV infection.

To date, ZIKV outbreaks have been documented in 89 countries and territories in Africa, the Americas, Asia, and the Pacific [4]. In 2007, there was an outbreak on the island of Yap [5], followed by an outbreak in 2013 in French Polynesia [6]. In 2015, the Americas experienced an outbreak of ZIKV for the first time, and the epidemic has been disseminated to more than 20 countries and territories in South, Central, and North America, and the Caribbean. Along with the circulation of ZIKV, clinicians reported an association between ZIKV infection and GBS in adults in July 2015 [7] and microcephaly in neonates in October 2015 [8]. Subsequent studies have revealed that ZIKV infection in pregnant women can lead to vertical transmission to approximately 20–30% of neonates [4], causing congenital abnormalities, including microcephaly and congenital eye disease [9,10,11,12]. This led the World Health Organization (WHO) to declare a public health emergency of international concern in February 2016.

In addition to transmission by mosquito bites, the sexual transmission of ZIKV has also been documented. This includes male-to-female [13,14,15], male-to-male [16], and suspected female-to-male transmission [17]. Other studies have demonstrated a persistent presence of ZIKV in semen [18,19,20,21,22], even several months after symptom onset [23,24,25,26]. The persistent presence of infectious ZIKV in bodily fluids may lead to a risk of sexual transmission. A previous study estimated that approximately 1% of ZIKV infections reported in Europe and the United States were acquired through sexual transmission [27], and the importance of sexual ZIKV transmission should be considered a serious concern.

The outbreak in Central and South American countries was finally contained for several reasons, including mosquito control and the development of herd immunity in these countries. Although herd immunity was likely achieved by asymptomatic ZIKV infection through mosquito bites, sexual transmission could also have a minor but significant role in the development of herd immunity. Therefore, the impact of ZIKV infection via the mucosal route on the induction of anti-ZIKV immunity remains to be elucidated.

In this study, we used a cynomolgus macaque model to investigate the impact of mucosal ZIKV infection on subcutaneous superchallenges. We intravaginally infected three macaques with the Asian ZIKV PRVABC59 strain for four successive days. Repeated intravaginal inoculation did not induce detectable viremia or other symptoms in these monkeys. Eleven days after the first intravaginal inoculation, three monkeys and four naïve monkeys were subcutaneously challenged with the homologous PRVABC59 strain. While all naïve monkeys developed viremia, the three intravaginally pre-infected monkeys completely suppressed viremia. A neutralizing assay demonstrated that repeated intravaginal inoculation resulted in the development of a potent neutralizing antibody. These results suggest that viral replication at mucosal sites can induce protective immunity without inducing systemic viremia or symptoms.

## 2. Results

### 2.1. Absence of Systemic Viremia and Clinical Symptoms in Cynomolgus Monkeys after Intravaginal Inoculation of ZIKV PRVABC59 Strain

**Figure 1:**
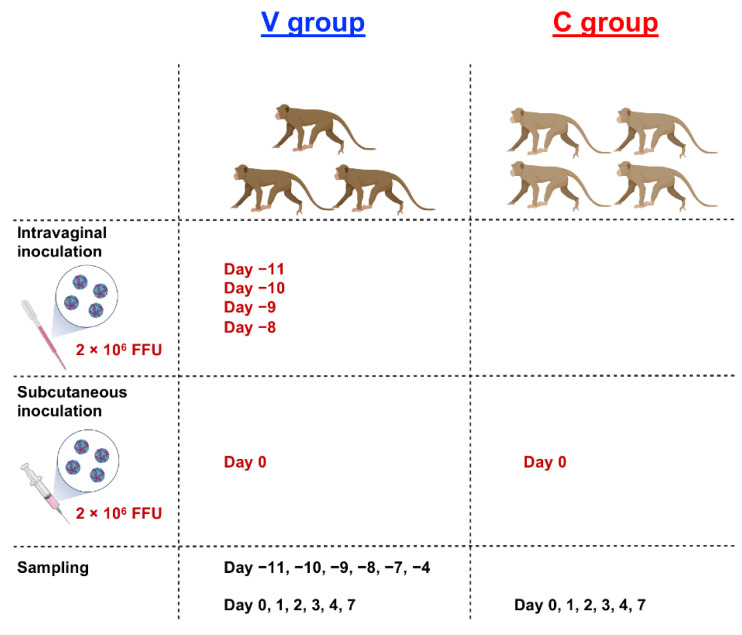
Schematic procedure of the in vivo experiment. Three female cynomolgus monkeys were intravaginally inoculated with the Zika virus (ZIKV) PRBABC59 strain on Days 8, 9, 10, and 11 (2 × 10^6^ focus forming units (FFU) at each time point) prior to subcutaneous infection. Seven female monkeys, including these three animals, were subcutaneously challenged with the ZIKV PRBABC59 strain (2 × 10^6^ FFU) on day zero.

**Figure 2:**
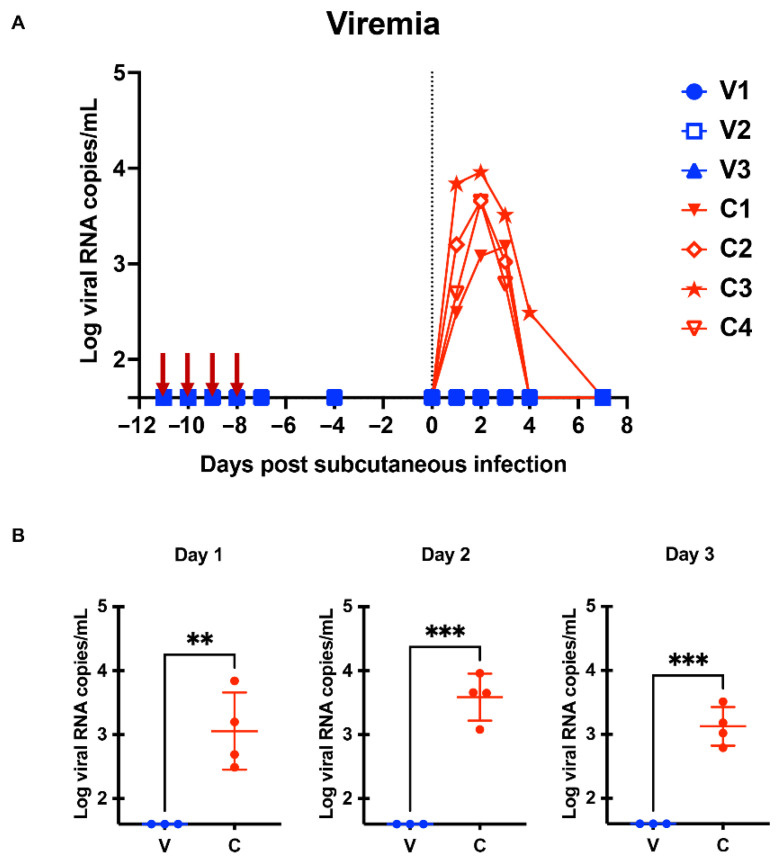
Plasma viral RNA load in cynomolgus monkeys. (**A**) Viral RNA levels in monkey plasma were quantified with quantitative real-time polymerase chain reaction (PCR). The red arrows indicate timepoints of intravaginal inoculation. While V1-V3 denotes each animal ID in group V (intravaginal pre-inoculation), C1-C4 denotes one in group C (control). (**B**) Differences in viral RNA levels on Days 1, 2, and 3 between groups V and C were examined by a two-tailed, unpaired Student *t*-test. *** *p* < 0.001, ** *p* < 0.01.

**Figure 3:**
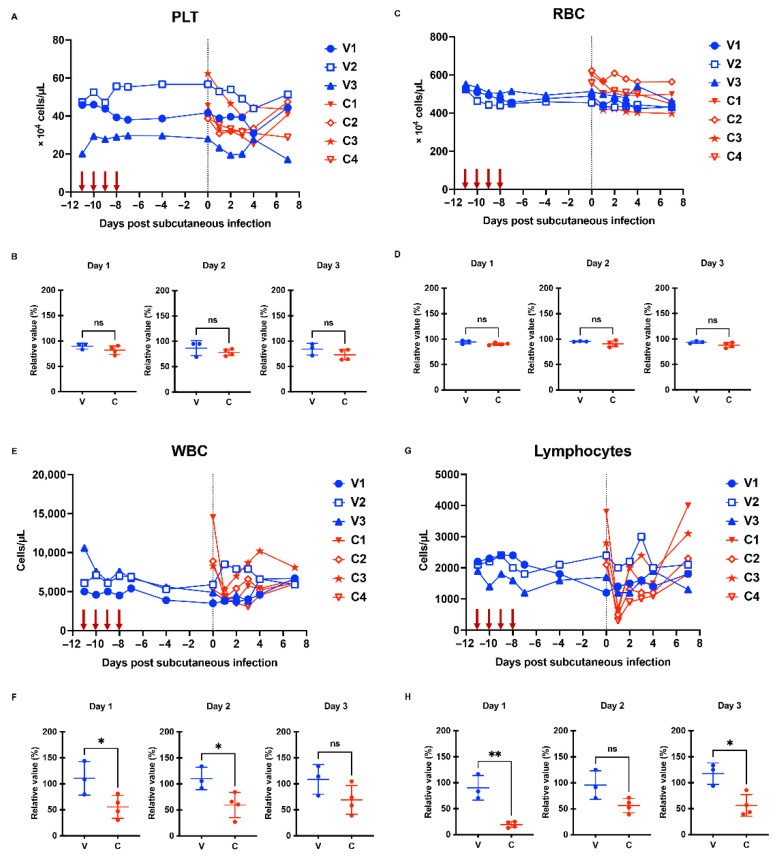
Changes in blood parameters in ZIKV-infected monkeys. (**A**) The number of platelets was counted with a hematology analyzer. While V1-V3 denotes each animal ID in group V (intravaginal pre-inoculation), C1-C4 denotes one in group C (control). (**B**) The relative value of platelets on Days 1, 2, and 3 was calculated compared to Day 0. Differences between groups V and C were examined by a two-tailed, unpaired Student *t*-test (ns, not significant). (**C**) The number of red blood cells (RBC) was counted with a hematology analyzer. (**D**) The relative value of red blood cells (RBC) on Days 1, 2, and 3 was calculated compared to Day 0. Differences between groups V and C were examined by a two-tailed, unpaired Student *t*-test (ns, not significant). (**E**) The number of white blood cells (WBC) was counted with a hematology analyzer. (**F**) The relative value of WBC on Days 1, 2, and 3 was calculated compared to Day 0. Differences between groups V and C were examined by a two-tailed, unpaired Student *t*-test. * *p* < 0.05, ns (not significant). (**G**) The number of lymphocytes was counted with a hematology analyzer. (**H**) The relative value of lymphocytes on Days 1, 2, and 3 was calculated compared to Day 0. Differences between groups V and C were examined by a two-tailed, unpaired Student *t*-test. ** *p* < 0.01, * *p* < 0.05, ns (not significant). ZIKV, Zika virus.

To investigate the immunological impact of intravaginal ZIKV infection, three monkeys were intravaginally inoculated with the PRVABC59 virus for four successive days to mimic repeated sexual intercourse (Figure 1). We used 2 × 10^6^ FFU for each inoculation based on a previous report [28]. We monitored plasma viral RNA levels and changes in blood parameters, including platelet (PLT), red blood cell (RBC), and white blood cell (WBC) counts. We observed that none of the monkeys developed detectable viremia 11 days after infection (Figure 2A). We observed a marginal change in blood cells, including PLT, RBC, and WBC counts (Figure 3A,C,E). No changes were observed in the skin of these macaques. This result suggests that the experimental protocol used in this study did not induce systemic viremia or symptoms in the cynomolgus macaques.

### 2.2. Monkeys Intravaginally Infected with the ZIKV PRVABC59 Strain Controlled the Subcutaneous Superchallenge with Homologous PRVABC59 Strain

**Figure 4:**
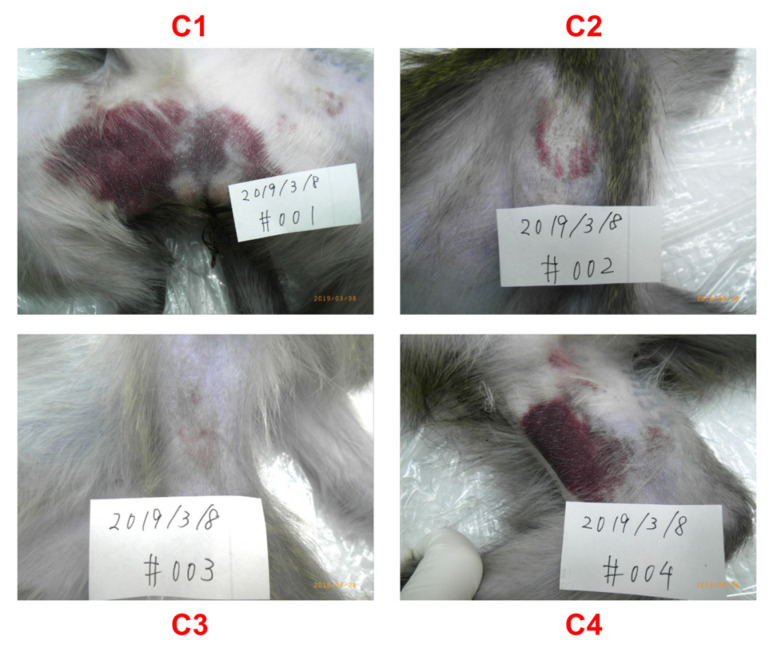
Change in the skin in ZIKV-infected monkeys. A change in the skin of group C (control) was observed. **C1**–**C4** denotes each animal ID in group C. ZIKV, Zika virus.

To test whether repeated intravaginal inoculation could induce protective immunity in three monkeys (hereafter referred to as the V group), we subcutaneously challenged these monkeys with the homologous PRVABC59 strain 11 days after the first intravaginal challenge. Four naïve macaques served as the control group (hereafter referred to as the C group) (Figure 1). We monitored the levels of plasma viral RNA load and changes in PLT, RBC, and WBC counts on Days 0, 1, 2, 3, 4, and 7. We observed a peak in viral RNA load between Days 2 and 3 in the C group. Similar to observations in a previous cynomolgus monkey model [29], the maximal viral load for monkeys in the C group was 1.5 × 10^3^–9.1 × 10^3^ genome copies/mL (Figure 2A). In sharp contrast, none of the monkeys in the V group showed detectable viremia (Figure 2A,B). Although we observed comparable changes in RBC between the V and C groups (Figure 3C,D), the C group tended to have a lower PLT level than the V group (Figure 3A,B). Notably, the C group had significantly lower WBC counts than the V group on Days 1 and 2 (Figure 3E,F). The C group showed a more significant decline in lymphocytes than the V group on Days 1 and 3 (Figure 3G,H). Flow cytometric analysis demonstrated that the decline in lymphocytes is associated with a rapid loss of T cell subsets (CD3^+^, CD16^−^ population) (Supplementary Figure S1). Furthermore, the C group exhibited apparent changes in the skin (Figure 4). We did not observe such changes in group V. We did not observe significant changes of body weight in both groups (Supplementary Figure S2A,B). Collectively, macaques in the V group were protected from the subcutaneous superchallenge.

### 2.3. Local Intravaginal Infection Induced a Potent Neutralizing Antibody

**Figure 5:**
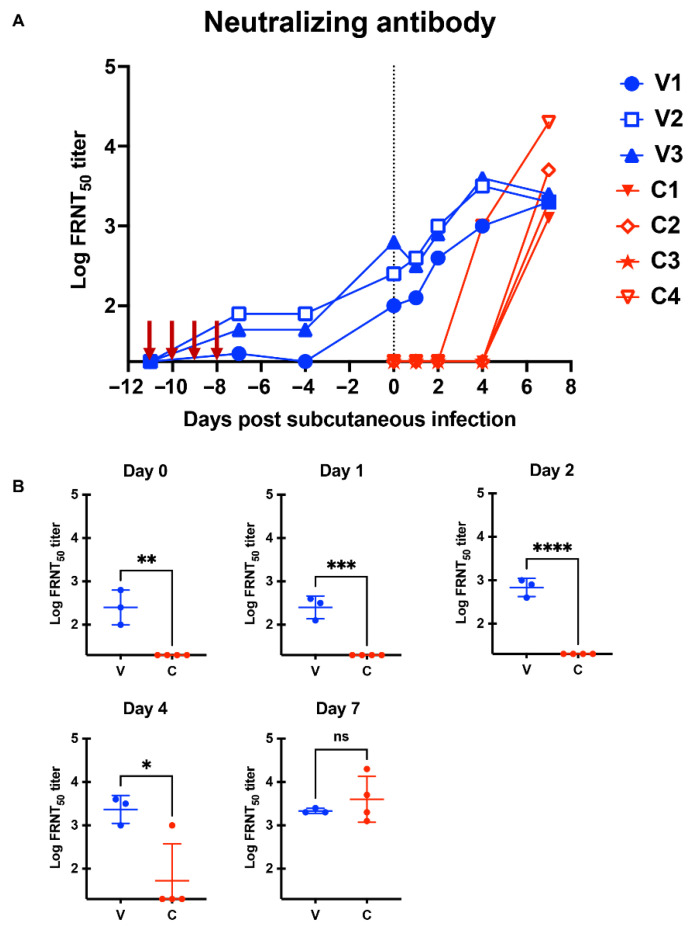
Monkeys in the V group developed a potent neutralizing antibody at the time of subcutaneous superchallenge. (**A**) The focus reduction neutralization test (FRNT)_50_ titers were calculated. While V1-V3 denotes each animal ID in group V (intravaginal pre-inoculation), C1-C4 denotes one in group C (control). (**B**) Differences in FRNT_50_ titers on Days 0, 1, 2, 4, and 7 between groups V and C were examined by a two-tailed, unpaired Student *t*-test. **** *p* < 0.0001, *** *p* < 0.001, ** *p* < 0.01, * *p* < 0.05, ns (not significant).

Next, we aimed to elucidate the underlying protective mechanisms. Therefore, we tested the induction of neutralizing antibodies in monkeys. First, we performed a focus reduction neutralization test (FRNT) on the homologous PRVABC59 strain. Monkeys in the V group developed >100 FRNT_50_ titers at the time of subcutaneous superchallenge (Figure 5A,B). As expected, monkeys in the C group had undetectable levels of neutralizing antibodies at the time of the subcutaneous challenge. After the subcutaneous challenge, monkeys in the V group had a much higher neutralizing antibody level compared to those in the C group. Next, to examine the broadness of neutralizing antibodies in the V group, we tested neutralizing activity against heterologous ZIKV strains, including three African strains (MR766-NIID, DAK AR 41524, and IbH 30656). The phylogenetic analysis of the E protein demonstrated that these strains were genetically distant from PRVABC59 (Supplementary Figure S3 and Table S1). The FRNT assay demonstrated that antibodies elicited in the V group blocked heterologous ZIKV strains (Supplementary Figure S4A,B). These results demonstrated that intravaginal ZIKV inoculation induced a potent neutralizing antibody that blocked a wide variety of ZIKV strains. Because previous studies demonstrated that ZIKV-binding IgM and IgG were rapidly induced in ZIKV-infected rhesus monkeys [30], we aimed to determine the class of immunoglobulins responsible for neutralization. Because protein G binds to human IgG but not IgM, we used a protein G column to isolate IgG from monkey plasma. The size of the purified protein on an SDS-PAGE gel was approximately 150 kDa (Supplementary Figure S5A), suggesting that the method used in this study specifically purified IgG from the plasma. The FRNT assay demonstrated that purified IgG (Post) showed a significantly lower FRNT_50_ titer than total plasma (Pre) against the PRVABC59 strains (Supplementary Figure S5B). This result showed that IgG had a limited role in neutralization in the V group, suggesting that immunoglobulin(s) other than IgG was (were) responsible for neutralization.

## 3. Discussion

In this study, we demonstrated that ZIKV infection at the mucosal site induced protective immunity and blocked subcutaneous superchallenge in a cynomolgus macaque model. Furthermore, this protection was associated with a neutralizing antibody at the time of subcutaneous challenge.

Mucosal ZIKV transmission should be prevented because there is a critical risk of fetal abnormalities occurring in pregnant women who are infected with ZIKV [4,9,10,11,12]. Approximately 10% of babies born to mothers with ZIKV infection have birth defects [31]. Notably, ZIKV infection during the first trimester is the most likely to cause birth defects [32,33,34,35]. The abnormalities induced by ZIKV infection have been reproduced in animal models. Intravaginal ZIKV infection in pregnant mice during early pregnancy leads to fetal growth restriction, infection of the fetal brain, and abortion, depending on the stage of pregnancy [36,37].

We showed that repeated intravaginal inoculation protected cynomolgus monkeys from viremia and the clinical symptoms induced by subcutaneous superchallenge (Figure 2, Figure 3F,H and Figure 4). Furthermore, intravaginal ZIKV infection was sufficient to induce neutralizing antibodies without systemic viremia (Figure 5A). A similar observation was reported in a study in which mice were intrarectally immunized with the ZIKV PRVABC59 strain [38]. Intrarectal inoculation of the PRVABC59 strain induced low viremia in *Ifnar1−/−* mice. In the subcutaneous superchallenge experiment, intrarectally inoculated mice were protected from infection 21 days after the intrarectal infection. This observation supports our finding that mucosal infections with limited viral replication can confer protective immunity. We further tested the cross-reactivity of plasma samples from the V group and showed that the plasma samples efficiently blocked not only the homologous PRVABC59 strain but also heterologous strains, including three African strains, MR766-NIID, DAK AR 41524, and IbH 30656 (Supplementary Figure S4A,B). These observations suggest that mucosal infection without systemic viremia or clinical symptoms can induce neutralizing antibodies that potently suppress broad ZIKV strains. Protection from subcutaneous superchallenge with a heterologous Asian ZIKV strain has also been observed in rhesus monkeys pre-inoculated with the African ZIKV strain via the subcutaneous route [39]. Notably, these monkeys had systemic viremia after the primary infection, suggesting that they developed stronger immunity against ZIKV than those in this study.

Moreover, an alarming observation was made. Owing to the co-circulation of ZIKV and DENV in endemic countries, cross-reactive antibodies may be produced, leading to a risk of antibody-dependent enhancement (ADE). A previous study demonstrated that immunodeficient AG129 mice administered monoclonal antibodies targeting ZIKV showed severe symptoms upon challenge with dengue virus type 2 (DENV-2) [40]. Similarly, a rhesus macaques pre-infected with ZIKV presented higher viremia and inflammatory response after DENV-2 challenge [41]. Therefore, the impact of mucosal ZIKV infection should be carefully evaluated. Future research should investigate whether the monkeys in this study developed an ADE antibody. Furthermore, the development of novel animal models using pigs [42], hamsters [43], guinea pigs [44,45], and tree shrews [46] can contribute to a better understanding of immunopathogenesis induced by ZIKV infection.

The ZIKV outbreak in Central and South America was controlled at the end of 2016. However, the reasons for this are not fully understood. Efforts to control the mosquito population likely contributed to the control of the epidemic. Nonetheless, the development of herd immunity in these regions could also be associated with containment (reviewed in [47]). A survey demonstrated that 63–73% of the population in Salvador, Brazil, was seropositive for ZIKV, leading to the development of herd immunity. This study suggested that herd immunity in this area contributed to the extinction of the ZIKV epidemic [48]. Another study demonstrated that pre-existing high-titer DENV antibodies limited the risk of ZIKV infection [49], suggesting that cross-reactive antibodies can also contribute to the dissemination of ZIKV in tropical and subtropical areas. Extensive surveys are necessary to monitor the seroprevalence of ZIKV and prevent future epidemics. Although the frequency of sexual ZIKV transmission is minor [27], the impact of mucosal ZIKV infection should also be considered.

The reason we were unable to induce systemic viremia in cynomolgus macaques upon successive intravaginal inoculations remains to be elucidated. One possible explanation is that the estrus cycle affected the outcome of the inoculations in these animals. The use of Depot-medroxyprogesterone acetate (DMPA) is associated with increased HIV susceptibility in women [50,51,52]. Furthermore, studies using rodent and macaque models have demonstrated that the condition of sexual hormones has a significant impact on susceptibility to viral infection, replication, and pathogenesis [53,54,55,56]. Previous studies using immunodeficient mice models suggested that the status of sex hormones can influence the permissiveness and persistence of ZIKV infection [57]. While mice infected during the estrus-like phase were resistant to intravaginal ZIKV infection, during the diestrus-like phase, they were susceptible. A similar observation was reported in a rhesus macaque model, where DMPA enhanced the susceptibility of the macaques to intravaginal infection [58]. Future research should address this issue since we did not synchronize the estrus cycle of monkeys in this study.

A limitation of this study is that the monkeys in the C group did not receive any treatment before the subcutaneous challenge. Nevertheless, our protocol for intravaginal inoculation did not include any surgical treatments. Therefore, the absence of pretreatment in the C group might not have had a significant impact on the outcome. In addition, because we were unable to test the viral shedding in body fluids and the changes in the blood chemistry test in infected monkeys, these points can be tested in a future study. The synchronization of the estrus cycle using a hormonal drug such as DMPA can be tested in a future study to elucidate the impact of the estrus cycle on susceptibility to ZIKV infection. Another limitation of this study is that we did not determine which immunoglobulin class was responsible for viral neutralization. However, it is reasonable to assume that IgM contributed to neutralization since purified IgG failed to neutralize ZIKV (Supplementary Figure S5A,B), and the neutralizing antibody was rapidly induced after intravaginal inoculation. This hypothesis is supported by a previous study in which ZIKV-binding IgM was rapidly induced after subcutaneous ZIKV infection [30]. Furthermore, because we immunized the V group via the intravaginal route, future research should examine whether intravaginally inoculated animals develop IgA with neutralizing activity in the plasma and secretions from mucosal sites.

In conclusion, our results revealed that mucosal ZIKV infection without systemic viremia and symptoms was sufficient to induce a potent neutralizing antibody, protecting macaques from subsequent subcutaneous superchallenge with homologous ZIKV strains. Furthermore, the antibody showed neutralizing activity against heterologous genetically distant ZIKV strains. These findings suggest a complex immunological event in individuals in epidemic areas of ZIKV, highlighting the potential of mucosal immunization to contain a ZIKV epidemic.

## 4. Materials and Methods

### 4.1. Ethics Statement

Animal experiments were carried out at the Tsukuba Primate Research Center, National Institutes of Biomedical Innovation, Health and Nutrition (NIBIOHN) (Ibaraki, Japan) with the help of HAMRI Co., Ltd. (Koga, Japan) for animal care and sample processing. All procedures were approved by the Committee on the Ethics of Animal Experiments of NIBIOHN (permission number: KAN30-05, and KAN31-04) and Osaka University (permission number: H30-03-0) under the guidelines for animal experiments at NIBIOHN, and Osaka University in accordance with the Guidelines for Proper Conduct of Animal Experiments established by the Science Council of Japan (http://www.scj.go.jp/ja/info/kohyo/pdf/kohyo-20-k16-2e.pdf) (accessed on 1 September 2022). The experiments were conducted in accordance with the “Weatherall report for the use of non-human primates in research” recommendations (https://royalsociety.org/topics-policy/publications/2006/weatherall-report/) (accessed on 1 September 2022). Animals were housed in adjoining individual primate cages, allowing them to make sight and sound contact with one another for social interactions, where the temperature was maintained at 25 °C with light for 12 h per day. The animals were fed apples and a commercial monkey diet (Type CMK-2; Clea Japan, Inc.) (Meguro, Japan). Blood collection and virus inoculation were performed under ketamine anesthesia. The animals were euthanized at the end of the experiment. At euthanasia, the animals were deeply anesthetized with pentobarbital under ketamine anesthesia, and whole blood was collected from the left ventricle.

### 4.2. Cell Culture

Vero cells (Japanese Collection of Research Bioresources Cell Bank (JCRB) (Ibaraki, Japan), Cat# JCRB9013) were cultured in modified Eagle’s medium (MEM, Nacalai Tesque, Cat# 21442-25), supplemented with 10% fetal bovine serum (FBS) (HyClone, Cat# SH30396), 1× non-essential amino acids solution (Nacalai Tesque, Cat# 06344-56), and 1× penicillin-streptomycin (P/S, Nacalai Tesque, Cat# 09367-34). C6/36 cells (JCRB, Cat# IFO50010) were cultured at 28 °C in Leibovitz L-15 medium (Thermo Fisher Scientific, Cat #11415064) supplemented with 10% FBS, 0.3% tryptose phosphate broth (Sigma-Aldrich, Cat# T8782-500G), and 1× P/S.

### 4.3. Viruses

The Asian strain of Zika virus (ZIKV), PRVABC59 (Human/2015/Puerto Rico) (NR-50240), an African ZIKV strain, the African ZIKV strain, DAK AR 41524 (Mosquito/1984/Senegal) (NR-50338), and IbH 30656 (Human/1968/Nigeria) (NR-50066) were obtained from Biodefense and Emerging Infections (BEI) Resources. The African strain of ZIKV, MR766-NIID (Rhesus/1947/Uganda), was obtained from the National Institute of Infectious Diseases, Japan (Shinjuku, Japan) [59]. The viruses were propagated in C6/36 cells. Viral titers were determined using a focus-forming assay in Vero cells.

### 4.4. Focus-Forming Assay

The virus stock was diluted 10-fold (1:10–1:10^6^) in FBS-free minimal essential medium (MEM). Diluted viruses (250 μL) were inoculated into the Vero cell monolayer in a 24-well plate and incubated at 37 °C for 2 h. The cells were overlaid with 500 μL MEM (Thermo Fisher Scientific, Cat #11935046) supplemented with 3% FBS and 1.5% carboxymethylcellulose sodium salt (Sigma-Aldrich, Cat# C4888-500G), and the plate was incubated at 37 °C for three days. The cells were washed three times with phosphate-buffered saline (PBS) (+) and fixed with 10% formaldehyde neutral buffer solution (Nacalai Tesque, Cat# 37152-51) for 20 min. After permeabilization with 1% Triton X-100 (Nacalai Tesque, Cat# 35501-15) in PBS (−) for 5 min, cells were incubated with mouse anti-flavivirus NS3 monoclonal antibody (34B1) [60] at 37 °C for 60 min. After washing with PBS (−), cells were incubated with goat anti-mouse IgG (H+L)-HRP (KPL, Cat# 074-1806) at 37 °C for 60 min. The foci of the infected cells were visualized using a Peroxidase Stain 3,3′-diaminobenzidine (DAB) Kit (Nacalai Tesque, Cat# 25985-50) prepared in the Metal Enhancer for DAB Stain (Nacalai Tesque, Cat# 07388-24).

### 4.5. Animal Experiment

Seven adult female cynomolgus monkeys were used in the present study. These animals were confirmed to be negative for pre-existing anti-ZIKV neutralizing antibodies. Three animals received repeated intravaginal inoculations of 2 × 10^6^ FFU of PRVABC59 on Day −11, −10, −9, and −8 prior to subcutaneous infection under ketamine-induced anesthesia. All animals were subcutaneously inoculated with 2 × 10^6^ focus forming units (FFU) of the PRVABC59 strain on day zero under ketamine anesthesia. Blood was collected on Days 0, 1, 2, 3, 4, and 7 under ketamine-induced anesthesia. The number of platelets (PLT), red blood cells (RBC), and white blood cells (WBC) was counted using a hematology analyzer (Sysmex).

### 4.6. Quantification of Plasma Viral RNA

Viral RNA was isolated from monkey plasma using a High Pure Viral RNA Kit (Roche, Cat# 11858882001), according to the manufacturer’s protocol. Viral RNA was quantified using a One Step TB Green PrimeScript PLUS reverse transcription polymerase chain reaction (RT-PCR) Kit (Perfect Real Time) (TaKaRa, Cat# RR096B) and the following primers for ZIKV: ZIKV F, 5’-AGGATCATAGGTGATGAAGAAAAGT-3´ and ZIKV R, 5´-CCTGACAACATTAAGATTGGTGC-3´ [61]. The total reaction volume was 12.5 µL per tube. The PCR conditions were 42 °C for 5 min and 95 °C for 10 s for reverse transcription, followed by 40 cycles at 95 °C for 5 s and 60 °C for 34 s. Fluorescent signals were detected using a QuantStudio 3 Real-Time PCR system (Thermo Fisher Scientific). The concentration of viral RNA (copies/mL) was determined by interpolation onto a standard curve of six 10-fold serial dilutions (4 × 10^6^ to 4 × 10^1^ copies/mL) of a synthetic ZIKV RNA fragment. The cutoff for the limit of detection of ZIKV RNA was 4 × 10^1^ copies/mL.

### 4.7. Focus Reduction Neutralization Test (FRNT)

Monkey plasma samples were heat-inactivated at 56 °C for 30 min and diluted four-fold (1:10 to 1:2560) in FBS-free MEM. Plasma samples (150 μL) were incubated with 100 FFU of PRVABC59, MR766-NIID, DAK AR 41524, or IbH 30656 (150 μL) at 37 °C for 1 h. Subsequently, the mixtures (250 μL) were transferred to a Vero cell monolayer in a 24-well plate and incubated at 37 °C for 2 h. Infected cells were quantified as described above. The neutralizing antibody titer was expressed as the maximum serum dilution, yielding a 50% reduction in the foci formed (FRNT_50_).

### 4.8. Immunophenotyping of Monkey Lymphocytes

Whole blood was labeled with antibodies against CD3 (clone SP34, APC/Cy7, BD Biosciences, Cat# 557757) and CD16 (clone 3G8, Brilliant Violet 711, BioLegend, Cat# 302044). After incubation, the blood samples were treated with fluorescence-activated cell sorting (FACS) Lysing Solution (BD Biosciences, Cat# 349202). Fluorescent signals were collected on an Attune NxT Flow Cytometer (Thermo Fisher Scientific) and analyzed using the FlowJo software (BD Biosciences).

### 4.9. Purification of IgG from Monkey Plasma Samples

IgG was purified from monkey plasma using IgG purification kit A (Dojindo, Cat# 349-91071). Equal volumes of plasma or purified IgG were mixed with 2× Bolt LDS sample buffer (Thermo Fisher Scientific, Cat# B0008) and incubated at 70 °C for 10 min. Proteins were separated on a Bolt 4–12% Bis-Tris Mini Protein Gel (Thermo Fisher Scientific, Cat # NW04122BOX) and visualized using CBB Stain One (Ready to Use) (Nacalai Tesque, Cat# 04543-51).

### 4.10. Construction of a Phylogenetic Tree

In total, 1029 complete amino acid sequences of the E protein from ZIKV strains are available in the Virus Pathogen Resource database (https://www.viprbrc.org/) (accessed on 29 September 2022). Among them, E protein sequences of 24 ZIKV strains were selected by redundancy removal using Jalview version 2.11.2.4 for the phylogenetic analysis, and the dengue virus type 2 (DENV-2) strain was used as the outgroup (Supplementary Figure S3). A phylogenetic tree was constructed using the maximum likelihood and the neighbor-joining method based on the Jones–Taylor–Thornton matrix-based model with 1000 bootstrap replicates using the MEGA X software.

### 4.11. Statistical Analysis

Differences in values between the animal groups were examined using an unpaired two-tailed Student’s *t*-test. Values with *p* < 0.05 or less were considered statistically significant. FRNT_50_ values were calculated using the Prism 9 software v9.1.1 (GraphPad Software) (San Diego, CA, USA).

## Data Availability

Not applicable.

## Supplementary Materials

The following supporting information can be downloaded at: https://www.mdpi.com/article/10.3390/ijms232214002/s1. Figure S1: Immunophenotyping of monkey lymphocytes; Figure S2: Changes in body weight of monkeys; Figure S3: Phylogenetic analysis of the ZIKV E protein; Figure S4: Neutralizing activity against heterologous ZIKV strains; Figure S5: IgG had a limited neutralizing activity; Table S1: Number of amino acid substitutions per site among the 24 ZIKV strains
