# Supplementary material for: Repeated Intravaginal Inoculation of Zika Virus Protects Cynomolgus Monkeys from Subcutaneous Superchallenge
Source: Int J Mol Sci. 2022 Nov 13;23(22):14002. doi: 10.3390/ijms232214002 (PMC9696507; doi:10.3390/ijms232214002)
Supplement: Supplementary file 1 [file ijms-23-14002-s001.zip › ijms-2004657-supplementary.pdf]

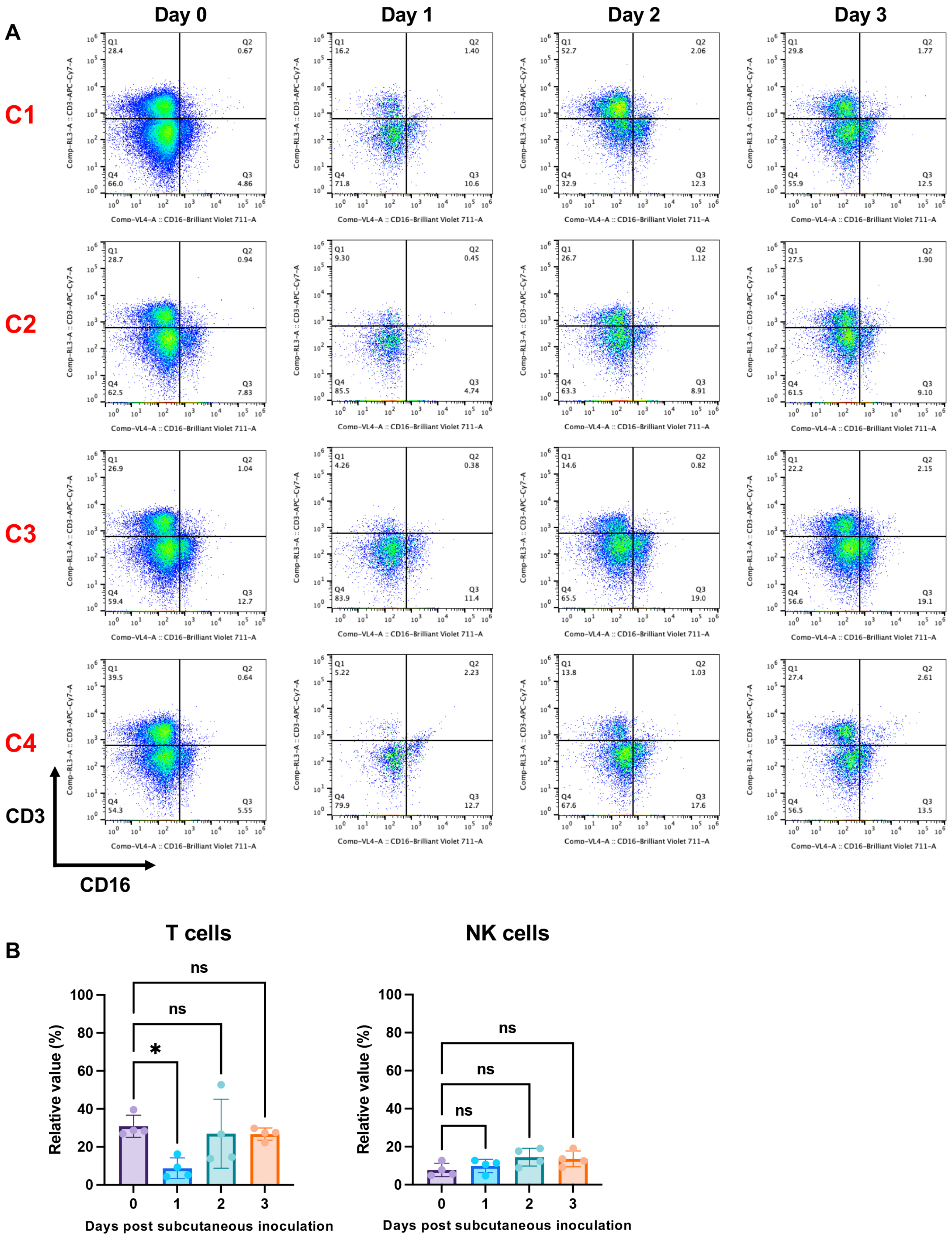

Supplementary Figure S1. Immunophenotyping of monkey lymphocytes.
(A) Blood of the C group collected at Day 0, 1, 2, and 3 after subcutaneous infection was stained with anti-CD3 and anti-CD16 antibodies. While $CD3^+$, $CD16^-$ population was classified as T cells, $CD3^-$, $CD16^+$ population was classified as NK cells. (B) Frequency of T cells or NK cells in the lymphocyte population was plotted. Differences between Day 0, 1, 2, and 3 were examined by one-way ANOVA, followed by the Tukey test. $^*p < 0.05$, ns (not significant). C1-C4 denotes each animal ID in group C.

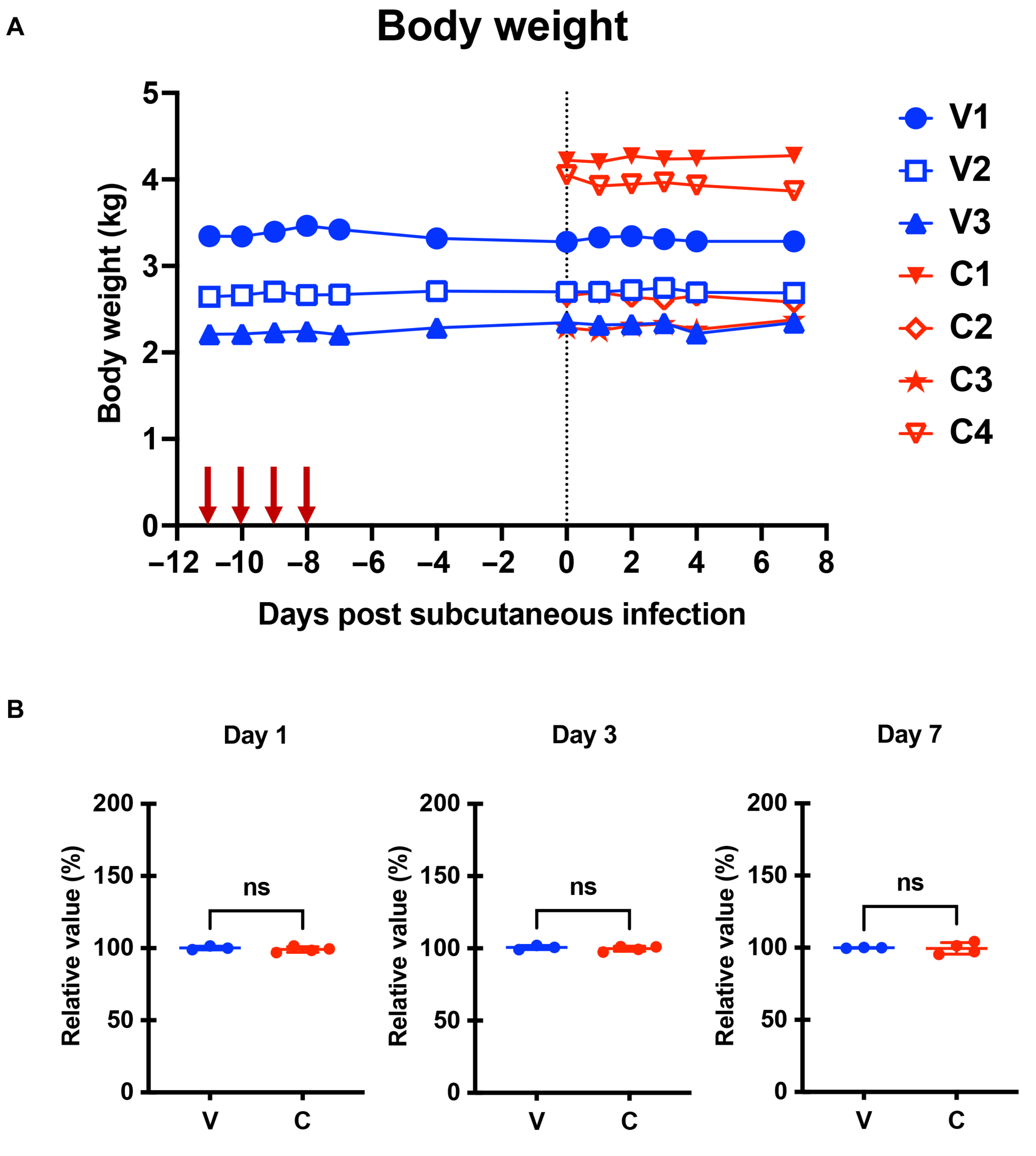

Supplementary Figure S2. Changes in body weight of monkeys.
(A) The number of white blood cells (WBC) was counted with a hematology analyzer. While V1-V3 denotes each animal ID in group V (intravaginal pre-inoculation), C1-C4 denotes one in group C (control). (B) The rel-ative value of WBC on Days 1, 2, and 7 was calculated compared to Day 0. Differences between groups V and C were examined by a two-tailed, unpaired Student t-test. * $p < 0.05$, ns (not signif-icant).

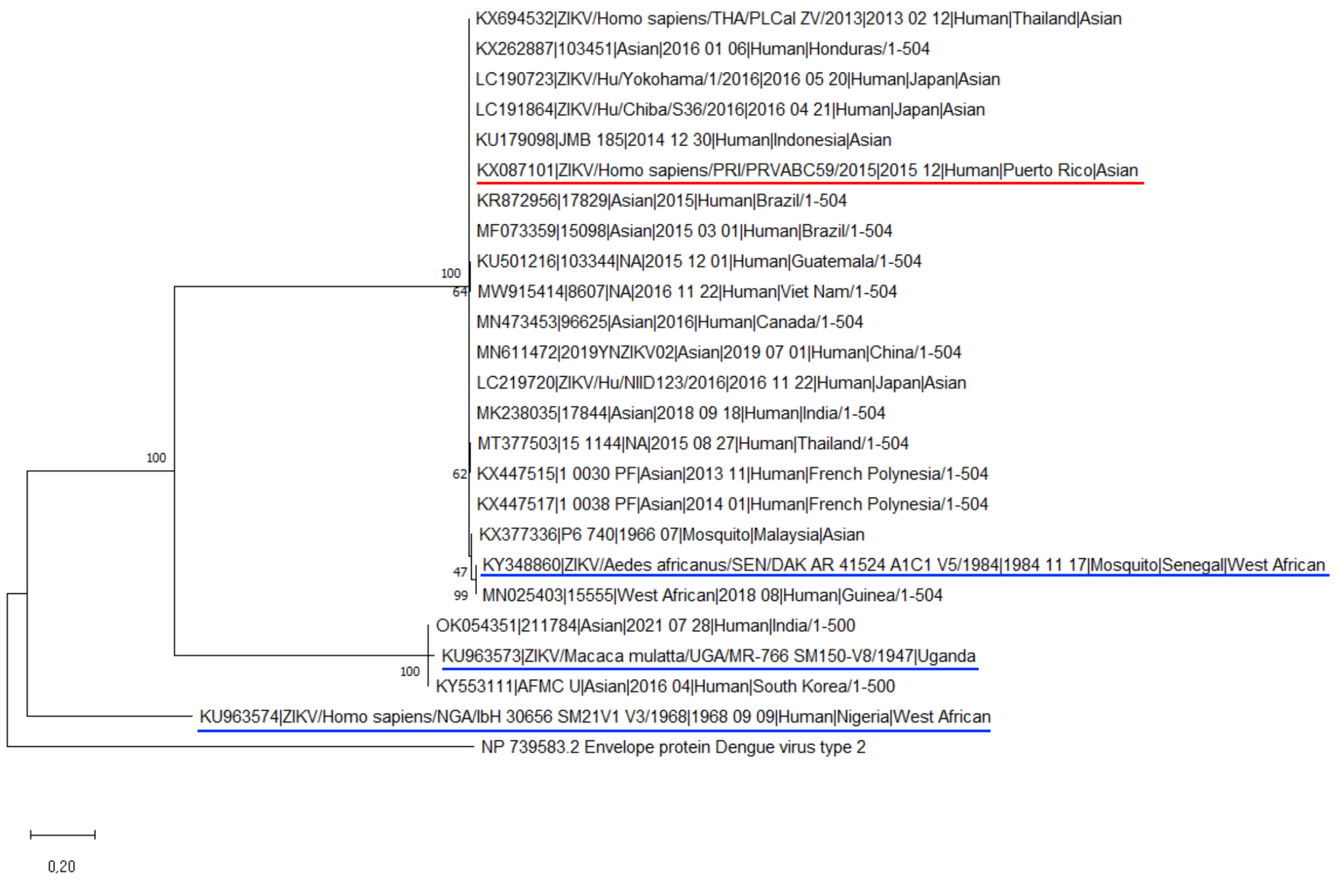

Supplementary Figure S3. Phylogenetic analysis of ZIKV E protein.
Phylogenetic tree of ZIKV E protein. E protein sequences of 24 ZIKV strains in the PDB database were used to construct a phylogenetic tree using maximum likelihood (ML) algorithm in MEGAX software. Note that PRVABC59 strain for in vivo experiment was marked with a red line, and three African strains (MR766-NIID strain, DAK AR 41524 strain and IbH 30656 strain) used for in vitro experiment were marked with blue lines. The numbers below and above the branch points denote the confidence levels of the relationship of the paired sequences determined by boot strap statistical analysis. The tree is drawn to scale, with branch lengths measured in the number of substitutions per site.

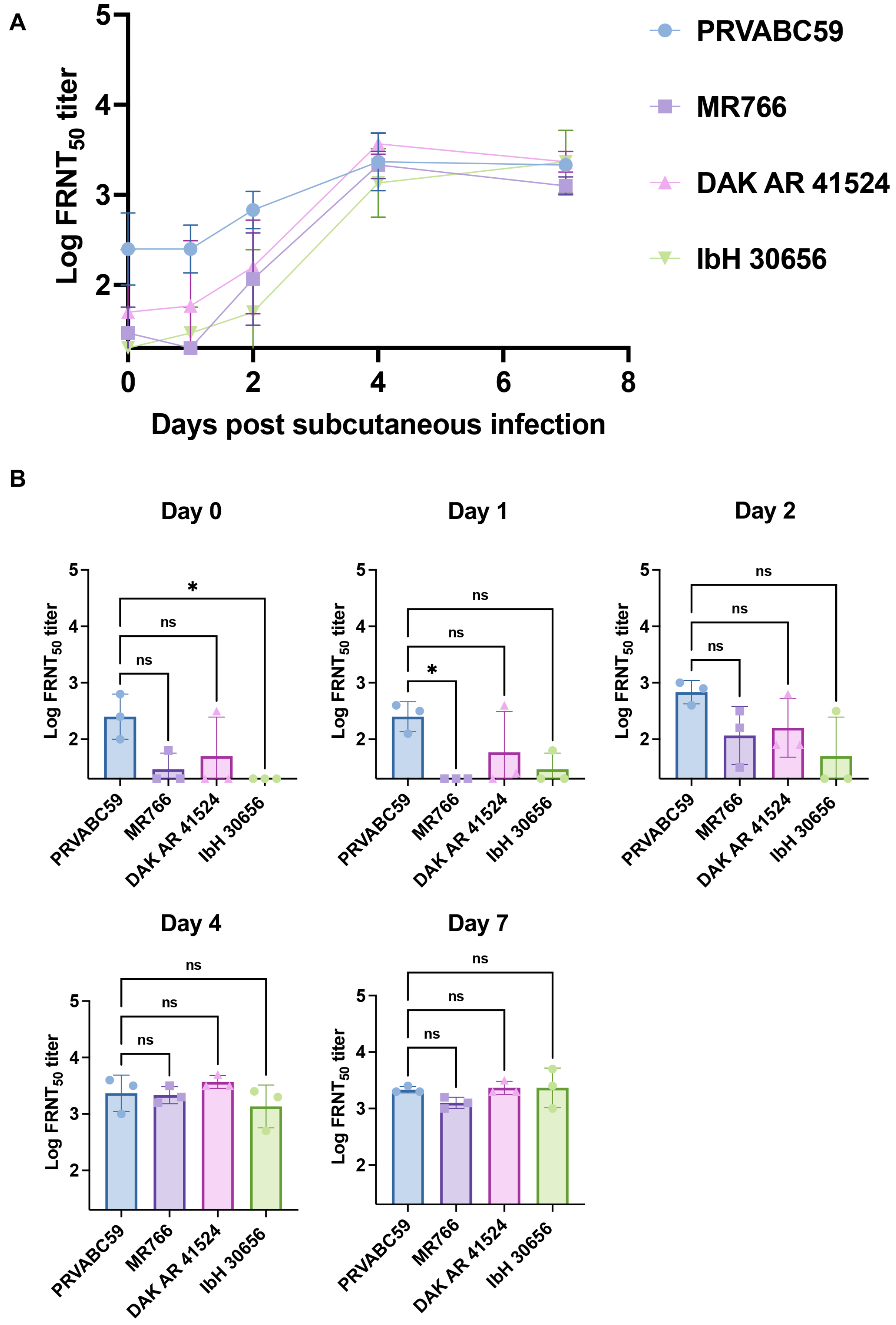

Supplementary Figure S4. Neutralizing activity against heterologous ZIKV strains.
(A) The FRNT50 values of group V (intravaginal pre-inoculation) against PRVABC59 strain, MR766-NIID strain, DAK AR 41524 strain and IbH 30656 strain were determined. (B) The FRNT50 values of the V group against PRVABC59 strain, MR766-NIID strain, DAK AR 41524 strain and IbH 30656 strain were compared at Day 0, 1, 2, 4 and 7 after subcutaneous infection. Differences were examined by one-way ANOVA, followed by the Tukey test. *$p < 0.05$, ns (not significant).

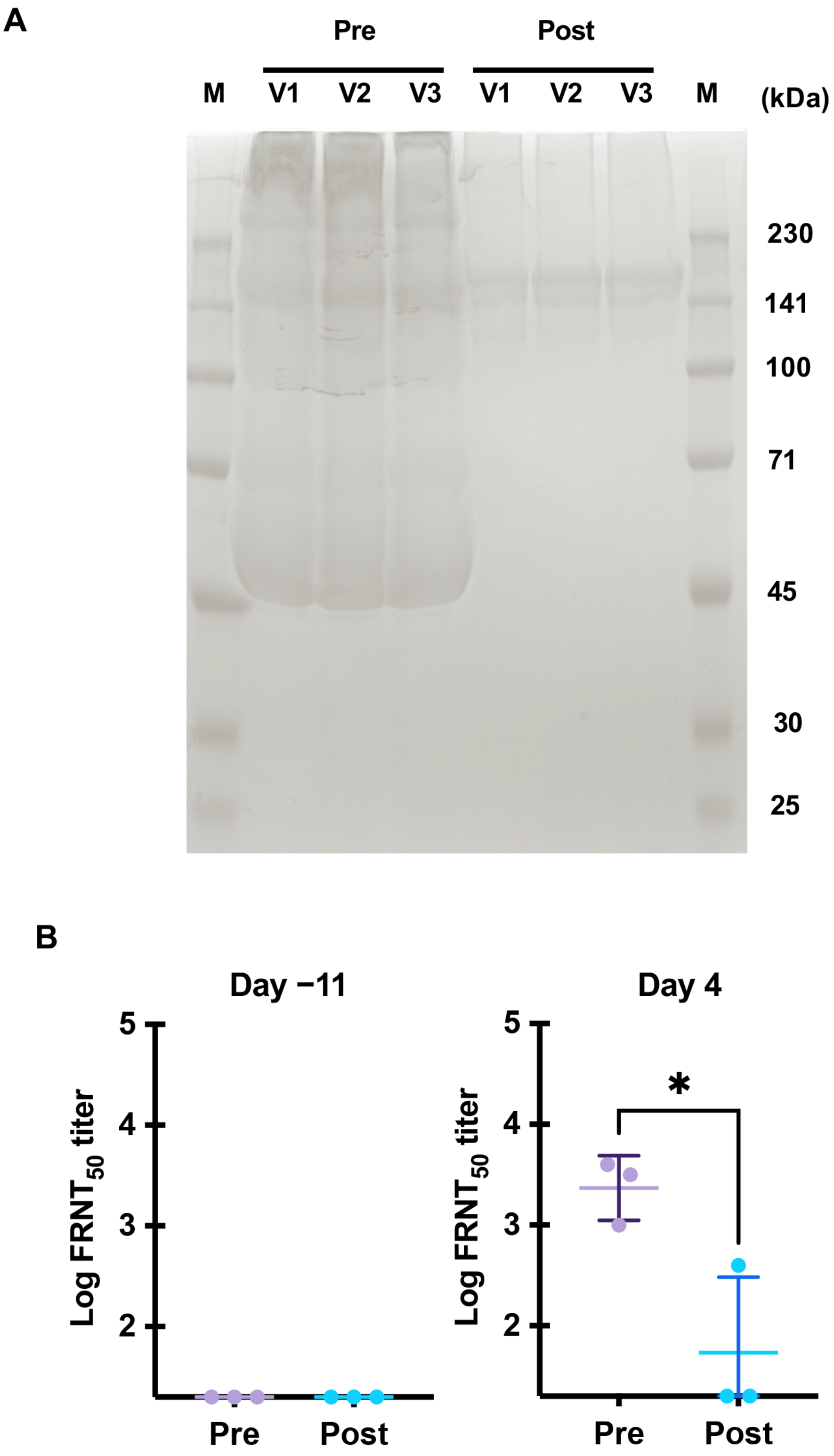

Supplementary Figure S5. IgG had a limited neutralizing activity.
(A) IgG was purified from plasma in using a protein G column. Plasma (Pre) and purified IgG were used for an SDS-PAGE analysis. The gel was stained with Coomassie Brilliant Blue. Plasma samples collected at Day 4 after subcutaneous infection were used. V1-V3 denotes each animal ID in group V (intravaginal pre-inoculation). (B) The $FRNT_{50}$ values of plasma (Pre) and the purified IgG (Post) against PRVABC59 strain was determined. Plasma samples collected at Day −11, and 4 after subcutaneous infection were used. Differences were examined by one-way ANOVA, followed by the Tukey test. $^*p < 0.05$, ns (not significant).

Supplementary Table S1. Number of amino acid substitutions per site among the 24 ZIKV strains

		1	2	3	4	5	6	7	8	9	10	11	12	13	14	15	16	17	18	19	20	21	22	23	24
1	KX087101|ZIKV/Homo_sapiens/PRI/PRVABC59/2015|Human|Puerto_Rico																								
2	KY348860|ZIKV/Aedes_africanus/SEN/DAK_AR_41524_A1C1_V5/1984|Mosquito|Senegal	0.02																							
3	KU963573|ZIKV/Macaca_mulatta/UGA/MR-766_SM150-V8/1947|Uganda	1.97	1.95																						
4	KU963574|ZIKV/Homo_sapiens/NGA/IbH_30656_SM21V1_V3/1968|Human|Nigeria	2.12	2.10	1.92																					
5	MN025403|15555|2018|Human|Guinea	0.02	0.00	1.95	2.10																				
6	KX694532|ZIKV/Homo_sapiens/THA/PLCal_ZV/2013|Human|Thailand	0.00	0.02	1.96	2.11	0.02																			
7	MT377503|15_1144|2015|Human|Thailand	0.01	0.02	1.97	2.12	0.02	0.00																		
8	KU179098|JMB_185|2014|Human|Indonesia	0.00	0.02	1.96	2.11	0.02	0.00	0.00																	
9	KX377336|P6_740|1966|Mosquito|Malaysia	0.01	0.02	1.98	2.11	0.02	0.01	0.01	0.01																
10	MW915414|8607|2016|Human|Viet_Nam	0.01	0.02	1.97	2.11	0.02	0.00	0.01	0.00	0.01															
11	LC191864|ZIKV/Hu/Chiba/S36/2016|2016|Human|Japan	0.00	0.02	1.96	2.11	0.02	0.00	0.00	0.00	0.01	0.00														
12	LC190723|ZIKV/Hu/Yokohama/1/2016|2016|Human|Japan	0.00	0.02	1.96	2.11	0.02	0.00	0.00	0.00	0.01	0.00	0.00													
13	LC219720|ZIKV/Hu/NIID123/2016|Human|Japan	0.01	0.02	1.97	2.11	0.02	0.00	0.01	0.00	0.01	0.01	0.00	0.00												
14	KY553111|AFMC_U|2016|Human|South_Korea	1.92	1.92	0.02	1.90	1.92	1.91	1.92	1.91	1.93	1.93	1.91	1.91	1.93											
15	OK054351|211784|2021|Human|India	1.93	1.93	0.03	1.91	1.93	1.92	1.93	1.92	1.94	1.93	1.92	1.92	1.93	0.01										
16	MK238035|17844|2018|Human|India	0.00	0.02	1.96	2.11	0.02	0.00	0.01	0.00	0.01	0.01	0.00	0.00	0.01	1.91	1.92									
17	MN611472|2019YNZIKV02|2019|Human|China	0.00	0.02	1.96	2.11	0.02	0.00	0.01	0.00	0.01	0.01	0.00	0.00	0.01	1.91	1.92	0.00								
18	MN473453|96625|2016|Human|Canada	0.00	0.02	1.97	2.11	0.02	0.00	0.01	0.00	0.01	0.01	0.00	0.00	0.01	1.92	1.93	0.00	0.00							
19	KX447515|1_0030_PF|2013|Human|French_Polynesia	0.00	0.02	1.96	2.11	0.02	0.00	0.00	0.00	0.01	0.01	0.00	0.00	0.01	1.92	1.92	0.00	0.00	0.00						
20	KX447517|1_0038_PF|2014_01|Human|French_Polynesia	0.01	0.02	1.95	2.12	0.02	0.00	0.01	0.00	0.01	0.01	0.00	0.00	0.01	1.90	1.91	0.01	0.01	0.01	0.01					
21	KU501216|103344|2015_12_01|Human|Guatemala	0.00	0.02	1.97	2.12	0.02	0.00	0.01	0.00	0.01	0.00	0.00	0.00	0.01	1.92	1.93	0.00	0.00	0.00	0.00	0.01				
22	KX262887|103451|2016_01_06|Human|Honduras	0.00	0.02	1.96	2.11	0.02	0.00	0.00	0.00	0.01	0.00	0.00	0.00	0.00	1.91	1.92	0.00	0.00	0.00	0.00	0.00	0.00			
23	MF073359|15098|2015_03_01|Human|Brazil	0.00	0.02	1.96	2.12	0.02	0.00	0.01	0.00	0.01	0.01	0.00	0.00	0.01	1.92	1.92	0.00	0.00	0.00	0.00	0.01	0.00	0.00		
24	KR872956|17829|2015|Human|Brazil	0.00	0.02	1.97	2.12	0.02	0.00	0.01	0.00	0.01	0.01	0.00	0.00	0.01	1.92	1.93	0.00	0.00	0.00	0.00	0.01	0.00	0.00	0.00	
